# Application of Genomic and Quantitative Genetic Tools to Identify Candidate Resistance Genes for Brown Rot Resistance in Peach

**DOI:** 10.1371/journal.pone.0078634

**Published:** 2013-11-11

**Authors:** Pedro J. Martínez-García, Dan E. Parfitt, Richard M. Bostock, Jonathan Fresnedo-Ramírez, Alejandra Vazquez-Lobo, Ebenezer A. Ogundiwin, Thomas M. Gradziel, Carlos H. Crisosto

**Affiliations:** 1 Department of Plant Sciences, University of California Davis, Davis, California, United States of America; 2 Department of Plant Pathology, University of California Davis, Davis, California, United States of America; 3 Departamento de Ecología Evolutiva, Instituto de Ecología, Universidad Nacional Autónoma de México, México DF, México; New Mexico State University, United States of America

## Abstract

The availability of a complete peach genome assembly and three different peach genome sequences created by our group provide new opportunities for application of genomic data and can improve the power of the classical Quantitative Trait Loci (QTL) approaches to identify candidate genes for peach disease resistance. Brown rot caused by *Monilinia* spp., is the most important fungal disease of stone fruits worldwide. Improved levels of peach fruit rot resistance have been identified in some cultivars and advanced selections developed in the UC Davis and USDA breeding programs. Whole genome sequencing of the Pop-DF parents lead to discovery of high-quality SNP markers for QTL genome scanning in this experimental population. Pop-DF created by crossing a brown rot moderately resistant cultivar ‘Dr. Davis’ and a brown rot resistant introgression line, ‘F8,1–42’, derived from an initial almond × peach interspecific hybrid, was evaluated for brown rot resistance in fruit of harvest maturity over three seasons. Using the SNP linkage map of Pop-DF and phenotypic data collected with inoculated fruit, a genome scan for QTL identified several SNP markers associated with brown rot resistance. Two of these QTLs were placed on linkage group 1, covering a large (physical) region on chromosome 1. The genome scan for QTL and SNP effects predicted several candidate genes associated with disease resistance responses in other host-pathogen systems. Two potential candidate genes, ppa011763m and ppa026453m, may be the genes primarily responsible for *M. fructicola* recognition in peach, activating both PAMP-triggered immunity (PTI) and effector-triggered immunity (ETI) responses. Our results provide a foundation for further genetic dissection, marker assisted breeding for brown rot resistance, and development of peach cultivars resistant to brown rot.

## Introduction

Brown rot caused by *Monilinia* spp., is the most important fungal disease of stone fruits worldwide [1]. Brown rot blossom blight and fruit rot can cause significant yield losses in most *Prunus* crop species. Brown rot is a serious problem for growers both before and after fruit harvest. Fruit susceptibility changes during the course of development; mature fruit are highly vulnerable to new infections and to the emergence of quiescent infections just prior to or following harvest. Worldwide, various plant pathogenic *Monilinia* species cause brown rot and some, such as *Monilinia fructicola* (G. Winter) Honey and *Monilinia fructigena* Honey, are quarantine pathogens because of the risks they pose to fruit production in regions where these aggressive species do not occur. In North America, *M. fructicola* and *Monilinia laxa* (Aderh. & Ruhland) Honey are the principal causal species of brown rot blossom blight and fruit rot in stone fruits.

The use of fungicides is the primary method to control brown rot in conventional production systems and, in the absence of fungicides, postharvest losses of peaches and nectarines can exceed 50% [2], [3] and approach 100% under conducive environmental conditions and high disease pressure. There is a strong desire within the industry and among consumers to reduce the use of chemical fungicides, as well as concern about the development of fungicide-resistance in pathogen populations [2], [4]. Consumer and regulatory demands for reduced chemical inputs in fruit crops underscore the need for alternative control measures. Biological control with microbial antagonists is being explored, but this approach has yet to show commercial feasibility. Thus, host-derived disease resistance presents a cost effective strategy, complementary to potential biological and existing cultural controls, and a sustainable alternative to help reduce the use of chemicals for disease management.

Commercial cultivars are generally susceptible to brown rot [5], [6]; however, improved levels of resistance have been identified in some cultivars such as the South American cv. ‘Bolinha’ [7]–[9]. Since 1994, over 4,000 peach genotypes representing landraces, standard canning peach cultivars, peach x almond hybrids, advanced experimental selections with various pedigrees including some with ‘Bolinha’ heritage, and progeny of mapping populations have been evaluated for mature fruit rot resistance to *M. fructicola* in controlled inoculations within the UC Davis cling peach breeding program [10]. These studies indicate wide variation in fruit rot resistance, suggesting multiple genes are functioning and that brown rot resistance can be modeled as a polygenic quantitative trait. Evaluation of 81 peach genotypes in which the fruit pericarp had been wounded or left unwounded at the site of inoculation indicated that brown rot resistance is associated with the pericarp (epidermal) or the mesocarp or both, depending on the genotype [11]. Subsequently, a preliminary QTL analysis was conducted on Pop-DF, a peach population derived from crosses between the susceptible cultivar ‘Dr. Davis’ and the resistant ‘F8,1-42’, a peach introgression line derived from an almond × peach interspecific hybrid. A total of 230 SSRs and 37 candidate gene (CG) primer pairs were screened for polymorphism using the Pop-DF parents and progeny, from which 52 SSR and two CGs were found to be polymorphic. The locations of putative QTLs conferring resistance to brown rot were placed on chromosome 1 after a comparison with the corresponding groups to the Texas × Early Gold peach reference map using common SSR markers.

The epidermal resistance, initially observed in mature fruit of cv. ‘Bolinha’ [2], [8], has provided a focus for our resistance screening program and for biochemical and cellular studies of the *M. fructicola*-peach interaction. Stone fruits become increasingly susceptible to pathogens as they mature and ripen, enabling quiescent infections to become active and new infections to progress [12]. Associated with this increased susceptibility are structural changes in the fruit surface, which includes thinning and fracturing of the cuticle, changes in fruit surface chemistry, such as production of sugars and a decline of phenolic compounds and organic acids, and changes in the structure and integrity of the fruit mesocarp [13]. *M. fructicola* responds to these changes by expressing genes and proteins that are important for the pathogen to successfully infect and colonize the fruit [14]. There are differences in fruit peel phenols among peach genotypes that differ in their resistance to the brown rot pathogen [15]. Prusky and Lichter [16] have reviewed pathogen quiescence in post-harvest diseases and discussed how fruit factors such as high acidity and phenols in unripe fruits can contribute to disease resistance. Thus, the multigenic nature of brown rot resistance, suggested by the genetic studies, is further supported by comparative studies of the biochemical and structural features associated with fruit of various genotypes and during development [13]. Possible mechanisms of resistance can be inferred from the identification of genes associated with disease resistance, allowing the incorporation of alleles for resistance into breeding programs with the use of linked markers.

High-throughput molecular genetic tools and a high-quality genome sequence [17] have been developed recently for peach (Peach v1.0), and can be applied to radically improve the efficiency of disease resistance breeding in peach. The new genomic technologies developed recently have reduced the cost and time to identify molecular genetic markers in plants by as much as 100-fold. In addition to the published peach genome sequence (Peach v1.0), three important peach cultivars were sequenced at UC Davis [18]–[20] and an important set of high-quality SNP makers obtained. This has permitted the development of new operational and statistical approaches to marker-assisted selection (MAS) and the application of markers in applied tree fruit breeding programs [18], [20]. In this paper we present results of a comprehensive study of QTLs for brown rot resistance in peach using the Pop-DF mapping population, and provide evidence for SNP markers tightly linked with brown rot resistance and predicted candidate resistance genes.

## Materials and Methods

### Mapping population

The SNP linkage map obtained for Pop-DF, a mapping populations from controlled crosses between the peach cultivar ‘Dr. Davis’ (female) and the almond x peach F_2_BC_2_ introgression line ‘F8, 1–42’, was used in this study [20]. ‘F8, 1–42’ has both almond (‘Nonpareil’) and peach (‘Jungerman’ and ‘Everts’) cultivars in its lineage, resulting in a unique phenotype that is freestone with non-melting flesh and high resistance to *M. fructicola*, which was probably inherited from almond. ‘Dr. Davis’ is a clingstone, non-melting, non-mealy, yellow flesh cultivar expressing low flesh browning potential and low levels of fruit aromatics, and is only moderately resistant to *M. fructicola* but has been the source of higher resistance in selfed progeny. The Pop-DF map covered 422 cM of the peach genome and included 1,037 SNP markers, with an average marker-site density of 1.48 cM/marker-site (and 0.41 cM/marker) [20].

### Genome sequencing and discovery and selection of SNPs

Three important peach cultivars, used as source of genetic diversity for the UC Davis Processing Peach Breeding Program, were sequenced using next generation sequencing technologies. A total of 11.3 Gb peach sequence and a set of 6,654 high-quality SNPs were obtained. All information about the SNP discovery, SNP names, SNP position, development of the Oligonucleotide Pool (OPA), Golden Gate Assay, SNP genotyping and map construction has been described in previous publications from our group [18]–[20].

### Disease assay and phenotypic data collection for Pop-DF

Fruit of similar size and maturity, based on visual assessment, were selected at random from trees at the UC Davis Pomology Orchards. Unblemished fruit from different individuals that had reached similar maturity in the Pop-DF population were collected. Twenty fruits from a single individual were collected at one time during each season, over a 6 to 7 week period throughout July and August, during the 2007–2009 seasons. Maturity differed slightly among seasons. Most of the progeny (∼80%) within the Pop-DF population were at harvest maturity during a three-week period in August and fruit from these individuals were evaluated during this period. Harvested fruit was stored at 4°C, for four days, until the day of the assay. Stored fruit was warmed to room temperature for 24 h prior to inoculation. Fruit was surface sterilized for 30 sec by immersion in 10% bleach (0.6% NaOCl), rinsed in deionized water, and dried. Approximately 20 unblemished fruits of each progeny were placed in humidified plastic containers (30.5 cm ×22.9 cm ×10.2 cm, Model 295C; Pioneer Plastics, Dixon, KY) with fruit tray liners (M-24B; FDS Manufacturing Co., Inc., Pomona, CA). The number of fruit tested varied depending upon the availability of fruit for each genotype, but typically 20 fruits per genotype were inoculated and evaluated for each trial. Each fruit was inoculated with a 10 μL droplet containing conidia of *M. fructicola* (isolate MUK-1; [13]) at a concentration of 2.5×10^4^ spores per mL from 7 to 10-day-old cultures maintained on V-8 juice agar. Controls were treated with a 10 μL droplet of water. All controls scored as 0 (no lesions). In addition to inoculations of non-wounded fruit (i.e., intact cuticle), parallel inoculations of wounded fruit were made by applying a 10 μL droplet of inoculum to a wound created by breaching the cuticle with a 22 gauge needle to generate a small hole to a depth of 2 mm. Controls were fruit with the wounds treated with a 10 μL droplet of water. Lesion diameter (mm) was recorded 3 days after inoculation and incubation of the peaches in the humidified containers at room temperature (2±1°C). Disease severity for each genotype was calculated as the product of the average lesion diameter x proportion of fruit with lesions greater than 3 mm (disease incidence). Standard cultivars (e.g., ‘Dr. Davis’, ‘Loadel’, ‘Ross’, or ‘Carson’) that are susceptible to brown rot were included each week, depending on their maturity and availability, to insure the presence of a positive control for each week's disease assay. Many genotypes within the Pop-DF population proved to be susceptible, especially after wound inoculation of the fruit, further affirming viability and pathogenicity of the inoculum. The data were collated and statistically analyzed using Microsoft Excel and JMP software version 7.0 [21].

Fruit color determinations, as an estimate of peach maturity were made immediately prior to inoculation. The method used throughout this study is a standard method we have used within the breeding program, which utilizes a handheld spectrophotometer (Konica-Minolta CM700) to measure peel color. Transmittance values for the visible spectrum (400–700 nm) were collected for each fruit and recorded. In addition, color photographs were taken with a digital camera for each genotype evaluated at the end of the disease assay and catalogued, providing a record for each individual fruit.

### QTL analysis of brown rot resistance in Pop-DF

QTL analysis of brown rot resistance was performed with MapQTL® 5.0 [22] using fruit disease severity data from each of the Pop-DF progeny obtained each growing season for the three seasons evaluated. Permutation tests and interval mapping (IM) were used to estimate the map position and the effect of each QTL. The likelihood value for presence of a QTL was expressed as a LOD score. The genome wide significance thresholds including all groups and individuals per linkage group were calculated with a 10,000-permutation test. The genome-wide significance thresholds at *P*-value 0.05 (or 5%) and 0.01 (or 1%) were used to detect significant QTLs or highly significant QTLs, respectively. The linkage-group-wide significance level of 5%, a less stringent threshold than used for previous analyses, was used to detect suggestive QTLs, i.e. QTLs that may be associated with disease phenotype but are not strongly supported statistically.

### Prediction of SNPs effects

A previous SNP effects database was created to locate each SNP within annotated transcripts or intronic regions using software SnpEff ver. 3.0c. [23] and reference to the ‘peach v1.0 genome’ sequence [19]. From this database, a specific list of genes and proteins associated with possible mechanisms of interest for brown rot resistance was assembled using statistically significant SNPs (nearest markers to QTLs for brown rot). The gene model for the peach genome annotation was generated using homology prediction with information publically available from several organisms and it is available at the Genome Database for Rosaceae (http://www.rosaceae.org/species/prunus_persica/genome_v1.0).

### Phyologenetic analysis

Phylogenetic analysis was carried out using a Neighbor-Joining method [24], and a 1000 replication bootstrap test for significance [25]. A Poisson model [26] was used for the amino acid substitution model implemented in Mega5 [27].

### Publicly available database

All sequences from Roche 454 and Illumina/Solexa have been submitted to NCBI for public use in the Short Read Archive database as ‘objects’ (search string “UC Davis peach”). SRA accession numbers are SRP003772 (‘Dr. Davis’), SRP003847 (‘F8, 1–42’), and SRP003848 (‘Georgia Belle’) in http://www.ncbi.nlm.nih.gov/sra?term=Sequence Read Archive. All SNPs have been deposited in the NCBI SNP database (dbSNP) at http://www.ncbi.nlm.nih.gov/SNP/snp_viewTable.cgi? handle = UCDAVISBIOINFO. The SNPs are in the range [NCBI-dbSNP:275372743 to NCBI-dbSNP:275395485].

## Results

### Screening for variation in fruit brown rot severity

**Figure 1:**
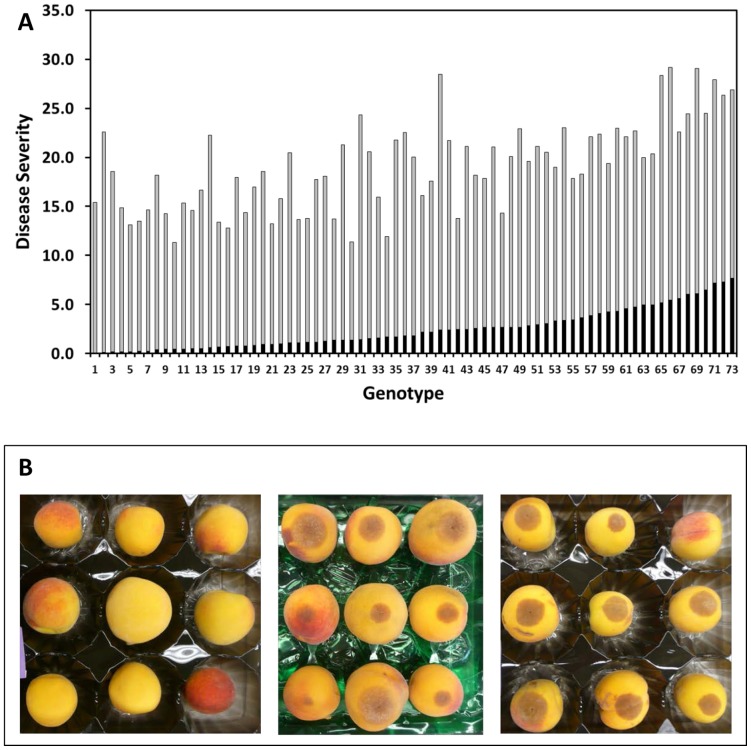
Disease analysis. Average disease severity for each of 73 genotypes within the PopDF population over three seasons (2007–2009) in order of increasing values for non-wounded fruit (black column) and the corresponding disease severity in wounded fruit for that genotype (gray column) (A). Representative fruit disease reactions of highly resistant (genotype 01,9–38; left panel) and highly susceptible (genotype 01,9–230; middle panel) peach genotypes in PopDF, 72 hours after inoculation with *Monilinia fructicola*. The average disease severity values corresponding to these sets of fruit are 0.0 (left) and 13.8 (middle). Right panel, disease reaction of wounded fruit of genotype 01,9–38. The average disease severity value corresponding to this set of fruit is 13.7 (B).

The highly virulent peach isolate MUK-1 was used throughout this study in controlled inoculations of fruit of harvest maturity to evaluate the fruit for their relative susceptibility to *M. fructicola*. The disease severity distribution of the non-wounded fruit when averaged across the three seasons of evaluation showed large differences in disease severity among individuals within the Pop-DF population (Figure 1 A and B). Reactions varied from near immunity (no lesions) to highly susceptible (>75% fruit with lesions). The fruit pictured in Figure 1B from the 2009 evaluation are illustrative of this range, and would correspond to disease severity values of 0.0 (left panel) and 13.8 (middle panel). Note that only genotypes for which we had data for all three seasons are presented in the disease severity distributions in Figure 1, and the values presented are averaged over the three years. For the averaged data, the highest disease severity value for non-wounded fruit was 7.7. The fruit of the progeny pictured were consistent in their ranking across the three seasons, although for most individuals within the population their relative rank in terms of resistance or susceptibility varied somewhat from year to year. Wounding the fruit generally abrogated any resistance to brown rot, and this is reflected in the disease severity distribution illustrated in Figure 1A. Even wounding and inoculation of fruit with very high cuticular/epidermal disease resistance generally resulted in a high disease severity rating and was comparable to the most susceptible lines (Figure 1B, right panel). Only two genotypes (genotypes 10 and 30 in Figure 1A) within the population consistently displayed strong epidermal and flesh resistance across the three seasons.

### QTL detection

**Figure 2:**
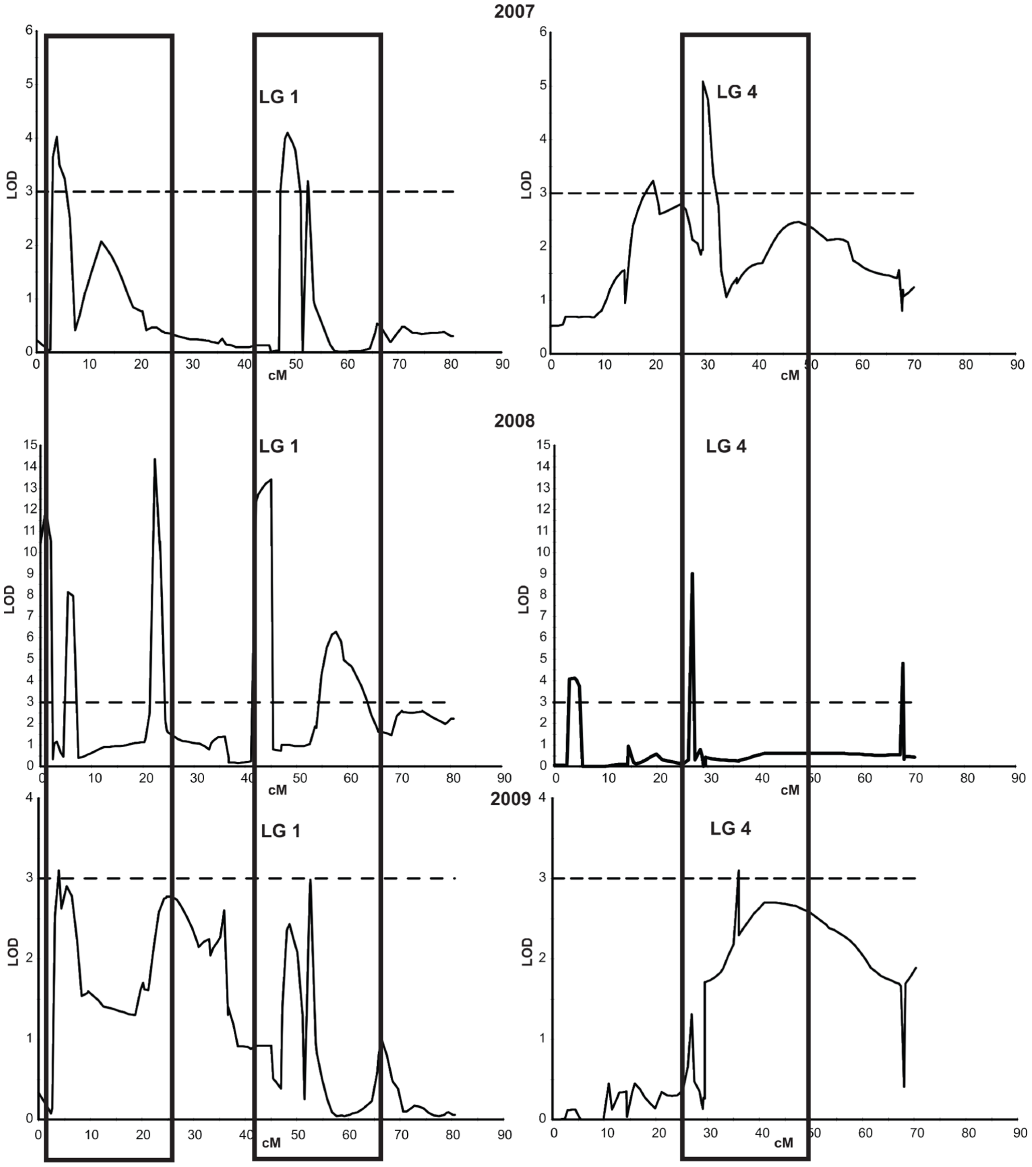
Description of the QTLs for the three years of evaluation. QTLs obtained for brown rot resistance in Pop-DF for three years in linkage group 1 (LG1) and linkage group 4 (LG4).

**Figure 3:**
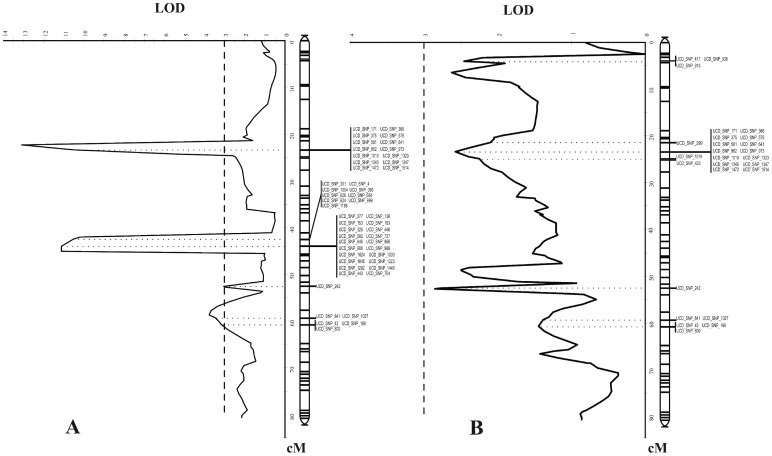
Comparison of candidate QTLs between years. QTLs on linkage group 1, using two(A) or three years data (B).

QTL detection was conducted using mean disease severity values for the genotypes in Pop-DF for the three years of evaluation, as well as by using disease severity values for each year of the study to calculate QTLs for individual years (Figure 2). A second approach used only values of the two first years of the study for a separate analysis (Figure 3). This data set was more complete than the composite data set for the three years because some genotypes could not be analyzed during 2009 due to powdery mildew infection and other factors that limited fruit availability for these genotypes.

**Figure 4:**
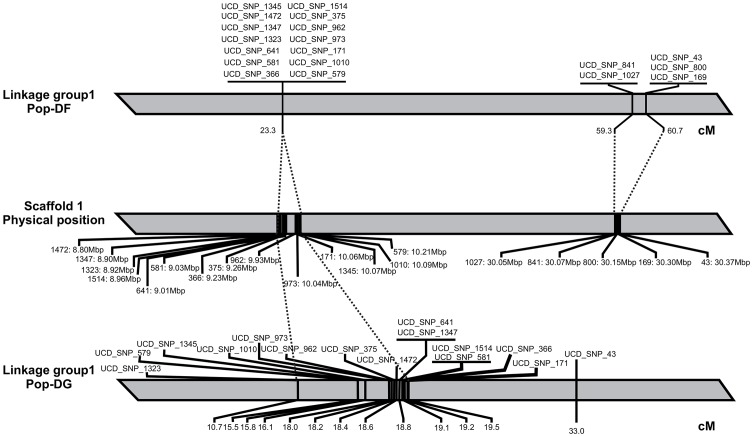
Physical map position of the QTLs. Comparison of genetic map positions of SNPs associated with Brown rot resistance on linkage group 1 of Pop-DF, with their physical map positions on chromosome 1 and their positions in linkage group 1 of Pop-DG.

The results for each year, taken separately, showed a consistent pattern for candidate QTLs across years, with two candidate QTLs (QTL1.1 and QTL1.2) in linkage group 1 (LG1) and one candidate QTL (QTL4) in linkage group 4 (LG4) associated with brown rot resistance (Figure 3). Although these candidate regions were not statistically significant for genome-wide association, they were significant for linkage-group-wide association with the three-year data set (Table 1). The QTL1.1 genetic interval covered about 20 cM (from 3.9 cM to 24.8 cM), corresponding to a physical distance of about 8.4 Mb. A set of 20 SNPs were significantly associated with QTL1.1, and explained between 19.9% and 44.4% of the phenotypic variance observed (Table 1). In this set, 14 SNPs were located within the same haplotype, representing a physical distance of about 1.41Mb (Figure 4).

**Table 1:** Identification of QTLs controlling brown rot resistance with three‘Dr. Davis’ x ‘F8-1,42’.

LG	SNP	LOD	LOD threshold		Position	R^2^
			Genome-wide	Linkage-group-wide		
1	UCD_SNP_417	2.46	4.5	2.7	3.90	44.30
1	UCD_SNP_538	2.46	4.5	2.7	3.91	44.40
1	UCD_SNP_913	2.46	4.5	2.7	3.91	44.40
1	UCD_SNP_299	2.1	4.5	2.7	21.33	19.90
1	UCD_SNP_171	2.57	4.5	2.7	23.34	26.60
1	UCD_SNP_366	2.57	4.5	2.7	23.34	26.60
1	UCD_SNP_375	2.57	4.5	2.7	23.34	26.60
1	UCD_SNP_579	2.57	4.5	2.7	23.34	26.60
1	UCD_SNP_581	2.57	4.5	2.7	23.34	26.60
1	UCD_SNP_641	2.57	4.5	2.7	23.34	26.60
1	UCD_SNP_962	2.57	4.5	2.7	23.34	26.60
1	UCD_SNP_973	2.57	4.5	2.7	23.34	26.60
1	UCD_SNP_1010	2.57	4.5	2.7	23.34	26.60
1	UCD_SNP_1323	2.57	4.5	2.7	23.34	26.60
1	UCD_SNP_1345	2.57	4.5	2.7	23.34	26.60
1	UCD_SNP_1347	2.57	4.5	2.7	23.34	26.60
1	UCD_SNP_1472	2.57	4.5	2.7	23.34	26.60
1	UCD_SNP_1514	2.57	4.5	2.7	23.34	26.60
1	UCD_SNP_1019	2.57	4.5	2.7	24.67	26.60
1	UCD_SNP_433	2.57	4.5	2.7	24.34	26.60
1	UCD_SNP_242	2.85	4.5	2.7	52.64	34.30
1	UCD_SNP_1027	1.39	4.5	2.7	59.28	14.20
1	UCD_SNP_841	1.39	4.5	2.7	59.28	14.20
1	UCD_SNP_43	1.45	4.5	2.7	60.72	15.10
1	UCD_SNP_169	1.45	4.5	2.7	60.72	15.10
1	UCD_SNP_800	1.45	4.5	2.7	60.72	15.10
4	UCD_SNP_770	1.24	4.5	2.7	29.48	62.30
4	UCD_SNP_493	0.99	4.5	2.7	33.07	60.30
4	UCD_SNP_546	1.1	4.5	2.7	36.02	32.90
4	UCD_SNP_273	1.12	4.5	2.7	36.04	58.80
4	UCD_SNP_1221	1.34	4.5	2.7	41.05	37.90
4	UCD_SNP_359	1.36	4.5	2.7	53.60	38.50
						

For each QTL detected, the linkage group, maximum LOD score, and percentage of variance explained (R2) are indicated.

QTL1.2 presented a genetic interval distance of about 25 cM, corresponding to a physical distance of about 18.9 Mb. QTL1.2 explained between 14.2% and 34.3% of the phenotypic variance observed. Six SNP markers were significantly associated with QTL1.2 (Table 1). SNP marker UCD_SNP_242 explained 34.3% of the phenotypic variance.

In the absence of significant statistical differences after interval mapping results, the presence of the QTL4 in relation to brown rot resistance cannot be confirmed (Table 1). The same QTL4 was not observed when only the 2007 and 2008 data representing a greater population size were analyzed together.

The same genetic and physical locations were observed for QTL1.1 and QTL1.2 using only the combined 2007 and 2008 data, but in this case the candidate regions were statistically significant for both genome-wide association and for linkage-group-wide association (Table 2). Together with the SNP markers associated with QTL1.2, two haplotypes were significantly associated with QTL1.2. These haplotypes explained approximately 76% of the phenotypic variance observed (Table 2).

**Table 2:** Identification of QTLs controlling brown rot resistance with two‘Dr. Davis’ x ‘F8-1,42’.

LG	SNP	LOD	LOD threshold		Position	R^2^
			Genome-wide	Linkage-group-wide		
1	UCD_SNP_171	10.42	4.5	2.7	23.34	70.10
1	UCD_SNP_366	10.42	4.5	2.7	23.34	70.10
1	UCD_SNP_375	10.42	4.5	2.7	23.34	70.10
1	UCD_SNP_579	10.42	4.5	2.7	23.34	70.10
1	UCD_SNP_581	10.42	4.5	2.7	23.34	70.10
1	UCD_SNP_641	10.42	4.5	2.7	23.34	70.10
1	UCD_SNP_962	10.42	4.5	2.7	23.34	70.10
1	UCD_SNP_973	10.42	4.5	2.7	23.34	70.10
1	UCD_SNP_1010	10.42	4.5	2.7	23.34	70.10
1	UCD_SNP_1323	10.42	4.5	2.7	23.34	70.10
1	UCD_SNP_1345	10.42	4.5	2.7	23.34	70.10
1	UCD_SNP_1347	10.42	4.5	2.7	23.34	70.10
1	UCD_SNP_1472	10.42	4.5	2.7	23.34	70.10
1	UCD_SNP_1514	10.42	4.5	2.7	23.34	70.10
1	UCD_SNP_301	10.54	4.5	2.7	42.53	76.30
1	UCD_SNP_4	10.54	4.5	2.7	42.53	76.30
1	UCD_SNP_1064	10.54	4.5	2.7	42.53	76.30
1	UCD_SNP_393	10.54	4.5	2.7	42.53	76.30
1	UCD_SNP_626	10.54	4.5	2.7	42.53	76.30
1	UCD_SNP_654	10.54	4.5	2.7	42.53	76.30
1	UCD_SNP_924	10.54	4.5	2.7	42.53	76.30
1	UCD_SNP_999	10.54	4.5	2.7	42.53	76.30
1	UCD_SNP_1188	10.54	4.5	2.7	42.53	76.30
1	UCD_SNP_377	11.14	4.5	2.7	44.03	76.60
1	UCD_SNP_136	11.14	4.5	2.7	44.03	76.60
1	UCD_SNP_153	11.14	4.5	2.7	44.03	76.60
1	UCD_SNP_183	11.14	4.5	2.7	44.03	76.60
1	UCD_SNP_326	11.14	4.5	2.7	44.03	76.60
1	UCD_SNP_446	11.14	4.5	2.7	44.03	76.60
1	UCD_SNP_502	11.14	4.5	2.7	44.03	76.60
1	UCD_SNP_737	11.14	4.5	2.7	44.03	76.60
1	UCD_SNP_846	11.14	4.5	2.7	44.03	76.60
1	UCD_SNP_860	11.14	4.5	2.7	44.03	76.60
1	UCD_SNP_906	11.14	4.5	2.7	44.03	76.60
1	UCD_SNP_988	11.14	4.5	2.7	44.03	76.60
1	UCD_SNP_1024	11.14	4.5	2.7	44.03	76.60
1	UCD_SNP_1030	11.14	4.5	2.7	44.03	76.60
1	UCD_SNP_1045	11.14	4.5	2.7	44.03	76.60
1	UCD_SNP_1223	11.14	4.5	2.7	44.03	76.60
1	UCD_SNP_1292	11.14	4.5	2.7	44.03	76.60
1	UCD_SNP_1446	11.14	4.5	2.7	44.03	76.60
1	UCD_SNP_1354	3.69	4.5	2.7	57.69	26.40
1	UCD_SNP_1027	3.48	4.5	2.7	59.28	25.30
1	UCD_SNP_841	3.48	4.5	2.7	59.28	25.40
1	UCD_SNP_43	3.18	4.5	2.7	60.72	24.30
1	UCD_SNP_169	3.18	4.5	2.7	60.72	24.30
1	UCD_SNP_800	3.18	4.5	2.7	60.72	24.30
4	UCD_SNP_89	4.3	4.5	2.7	2.94	46.70
4	UCD_SNP_324	4.3	4.5	2.7	2.94	46.70
4	UCD_SNP_1137	4.3	4.5	2.7	2.94	46.70
4	UCD_SNP_1488	4.3	4.5	2.7	2.94	46.70
4	UCD_SNP_772	4.17	4.5	2.7	4.40	46.30
4	UCD_SNP_275	4.2	4.5	2.7	4.88	45.50

For each QTL detected, the linkage group, maximum LOD score, and percentage of variance explained (R2) are indicated.

### Proteins associated with candidate SNPs related to brown rot resistance

A list of 26 SNP markers, associated with brown rot resistance, was selected to predict the expected effects of changes to these SNPs on annotated genes within the peach genome. There are a number of functionally interesting proteins predicted by this analysis, and some of these may be of mechanistic interest for understanding the plant-microbe interaction for brown rot and other diseases of peach. These include a senescence-associated related protein (UCD_SNP_171), a hypersensitive response-associated protein (UCD_SNP_641), a phosphomannomutase/phosphoglucomutase (UCD_SNP_169), a putative pectin esterase inhibitor 28 (UCD_SNP_366), a multidrug and toxin extrusion protein 1 (UCD_SNP_973), a MLP-like protein 329 (UCD_SNP_800), a bidirectional sugar transporter SWEET8 (UCD_SNP_1010), a mitogen-activated protein kinase 12 (UCD_SNP_1027), and a receptor-like protein kinase ANXUR2 (UCD_SNP_1472) (Table 3).

**Table 3:** The predicted effects of each SNP associated with brown rot resistance.

SNPname	Effect	Homologous gene	Reference Organism	Description
UCD_SNP_43 (scaffold 1:30378223)	UPSTREAM: 1868 bases	PGMC_POPTN	*Populus tremula*	Phosphoglucomutase, cytoplasmic
UCD_SNP_169 (scaffold 1:30300293)	UPSTREAM: 985 bases	AT1G70780.1	*Arabidopsis thaliana*	unknown protein
UCD_SNP_169 (scaffold 1:30300293)	UPSTREAM: 3172 bases	ALGC_PSESM	*Pseudomonas syringae pv. tomato*	Phosphomannomutase/phosphoglu comutase
UCD_SNP_169 (scaffold 1:30300293)	UPSTREAM: 1661 bases	AGD12_ARATH	*Arabidopsis thaliana*	ADP-ribosylation factor GTPase-activating protein AGD12
UCD_SNP_171 (scaffold 1:10062128)	DOWNSTREAM: 3223 bases	FANCI_HUMAN	*Homo sapiens*	Fanconi anemia group I protein
UCD_SNP_171 (scaffold 1:10062128)	UTR_3_PRIME: 29 bases from CDS	DYL2_RAT	*Rattus norvegicus*	Dynein light chain 2, cytoplasmic
UCD_SNP_171 (scaffold 1:10062128)	DOWNSTREAM: 4733 bases	AT5G49120.1	*Arabidopsis thaliana*	senescence-associated protein-related
UCD_SNP_242 (scaffold 1:25996225)	INTERGENIC			
UCD_SNP_299 (scaffold 1:7853986)	INTRON	AB11B_ARATH	*Arabidopsis thaliana*	ABC transporter B family member 11
UCD_SNP_366 (scaffold 1:9237272)	UPSTREAM: 3589 bases	N/A	N/A	N/A
UCD_SNP_366 (scaffold 1:9237272)	DOWNSTREAM: 592 bases	PME28_ARATH	*Arabidopsis thaliana*	Putative pectinesterase/pectinesterase inhibitor 28
UCD_SNP_375 (scaffold 1:9264458)	UPSTREAM: 3498 bases	AT1G21280.1	*Arabidopsis thaliana*	unknown protein
UCD_SNP_417 (scaffold 1:1848507)	UPSTREAM: 2766 bases	PP250_ARATH	*Arabidopsis thaliana*	Pentatricopeptide repeat-containing protein At3g23020
UCD_SNP_417 (scaffold 1:1848507)	DOWNSTREAM: 426 bases	APEH_MOUSE	*Mus musculus*	Acylamino-acid-releasing enzyme
UCD_SNP_433 (scaffold 1:10285225)	INTRON	CARMB_ARATH	*Arabidopsis thaliana*	Probable histone-arginine methyltransferase CARM1B
UCD_SNP_433 (scaffold 1:10285225)	INTRON	CARMB_ARATH	*Arabidopsis thaliana*	Probable histone-arginine methyltransferase CARM1B
UCD_SNP_433 (scaffold 1:10285225)	DOWNSTREAM: 3884 bases	GT2_ARATH	*Arabidopsis thaliana*	Putative glycosyltransferase 2
UCD_SNP_538 (scaffold 1: 1720528)	DOWNSTREAM: 955 bases	AT3G22970.1	*Arabidopsis thaliana*	unknown protein
UCD_SNP_579 (scaffold 1:10211076)	INTRON	KIF2C_MOUSE	*Mus musculus*	Kinesin-like protein KIF2C
UCD_SNP_581 (scaffold 1:9033319)	DOWNSTREAM: 3248 bases	N/A	N/A	N/A
UCD_SNP_641 (scaffold 1:9010281)	DOWNSTREAM: 2537 bases	TMVRN_NICGU	*Nicotiana glutinosa*	TMV resistance protein N
UCD_SNP_641 (scaffold 1:9010281)	UPSTREAM: 16 bases	N/A	N/A	N/A
UCD_SNP_800 (scaffold 1:30152386)	UPSTREAM: 432 bases	ML329_ARATH	*Arabidopsis thaliana*	MLP-like protein 329
UCD_SNP_800 (scaffold 1:30152386)	DOWNSTREAM: 3037 bases	ML329_ARATH	*Arabidopsis thaliana*	MLP-like protein 329
UCD_SNP_841 (scaffold 1:30070766)	UPSTREAM: 4918 bases	NUD25_ARATH	*Arabidopsis thaliana*	Nudix hydrolase 25
UCD_SNP_841 (scaffold 1:30070766)	SYNONYMOUS_CODING PAC:17646556	UCKC_DICDI	*Dictyostelium discoideum*	Uridine-cytidine kinase C
UCD_SNP_841 (scaffold 1:30070766)	SYNONYMOUS_CODING PAC:17646557	UCKC_DICDI	*Dictyostelium discoideum*	Uridine-cytidine kinase C
UCD_SNP_913 (scaffold 1:1766173)	DOWNSTREAM: 4323 bases	AT3G22990.1	*Arabidopsis thaliana*	LFR (LEAF AND FLOWER RELATED); binding
UCD_SNP_913 (scaffold 1:1766173)	UPSTREAM: 717 bases	KCS17_ARATH	*Arabidopsis thaliana*	3-ketoacyl-CoA synthase 17
UCD_SNP_962 (scaffold 1:9934122)	DOWNSTREAM: 3407 bases	ACD10_MOUSE	*Mus musculus* (Mouse)	Acyl-CoA dehydrogenase family member 10
UCD_SNP_962 (scaffold 1:9934122)	NON_SYNONYMOUS_CODING	PME28_ARATH	*Arabidopsis thaliana*	Putative pectinesterase/pectinesterase inhibitor 28
UCD_SNP_962 (scaffold 1:9934122)	UPSTREAM: 4029 bases	PME28_ARATH	*Arabidopsis thaliana*	Putative pectinesterase/pectinesterase inhibitor 28
UCD_SNP_973 (scaffold 1:10047284)	DOWNSTREAM: 391 bases	S47A1_XENTR	*Xenopus tropicalis*	Multidrug and toxin extrusion protein 1
UCD_SNP_1010 (scaffold 1:10096628)	UPSTREAM: 4700 bases	AT5G49100.1	*Arabidopsis thaliana*	unknown protein
UCD_SNP_1010 (scaffold 1:10096628)	DOWNSTREAM: 507 bases	RPG1_ARATH	*Arabidopsis thaliana*	Protein RUPTURED POLLEN GRAIN 1
UCD_SNP_1019 (scaffold 1:7055052)	UPSTREAM: 4182 bases	CLPB1_SYNY3	*Synechocystis sp.*	Chaperone protein clpB 1
UCD_SNP_1027 (scaffold 1:30052203)	DOWNSTREAM: 3014 bases	MPK12_ORYSJ	*Oryza sativa subsp. japonica*	Mitogen-activated protein kinase 12
UCD_SNP_1027 (scaffold 1:30052203)	DOWNSTREAM: 3014 bases	MPK12_ORYSJ	*Oryza sativa subsp. japonica*	Mitogen-activated protein kinase 12
UCD_SNP_1027 (scaffold 1:30052203)	DOWNSTREAM: 3014 bases	MPK12_ORYSJ	*Oryza sativa subsp. japonica*	Mitogen-activated protein kinase 12
UCD_SNP_1027 (scaffold 1:30052203)	UPSTREAM: 1740 bases	AT1G14990.1	*Arabidopsis thaliana*	unknown protein
UCD_SNP_1027 (scaffold 1:30052203)	UPSTREAM: 1483 bases	AT1G14990.1	*Arabidopsis thaliana*	unknown protein
UCD_SNP_1323 (scaffold 1:8921890)	INTERGENIC			
UCD_SNP_1345 (scaffold 1:10070654)	INTRON	FANCI_HUMAN	*Homo sapiens*	Fanconi anemia group I protein
UCD_SNP_1345 (scaffold 1:10070654)	UPSTREAM: 2973 bases	MFPA_CUCSA	*Cucumis sativus*	Glyoxysomal fatty acid beta-oxidation multifunctional protein MFP-a
UCD_SNP_1347 (scaffold 1:8900413)	INTERGENIC			
UCD_SNP_1472 (scaffold 1:8809914)	UPSTREAM: 2776 bases	ANX2_ARATH	*Arabidopsis thaliana*	Receptor-like protein kinase ANXUR2
UCD_SNP_1472 (scaffold 1:8809914)	SYNONYMOUS_CODING	ANX2_ARATH	*Arabidopsis thaliana*	Receptor-like protein kinase ANXUR2
UCD_SNP_1514 (scaffold 1:8965203)	INTERGENIC			

The homologous gene, the reference organism and description of the protein, associated with known protein coding regions are shown for each SNP.

## Discussion

The high-density SNP map obtained for Pop-DF combined with the disease severity data allowed a preliminary analysis of the genetic basis of the epidermal-associated resistance to brown rot disease in peach fruit. Our strategy, which incorporated disease phenotyping of harvested fruit with similar maturity under controlled laboratory conditions, was designed to minimize nongenetic variation in the QTL analysis from experimental errors and environmental factors.

Despite the lack of statistically significant differences in the QTL detection in our study, (likely due principally to the small population available for evaluation), the combination of QTL detection and SNP map-based gene prediction identified a set of candidate genes associated with brown rot resistance in peach with a high confidence (Table 3). These candidate genes are located on the physical genome (peach v1.0), close to a QTL controlling the disease phenotype. Despite the large genetic distance observed for each QTL and the high linkage disequilibrium (LD) observed in peach, extending up to 13–15 cM [28], our results from genome scanning found candidate genes with the predicted SNP effects strongly correlated with the disease response in peach.

**Figure 5:**
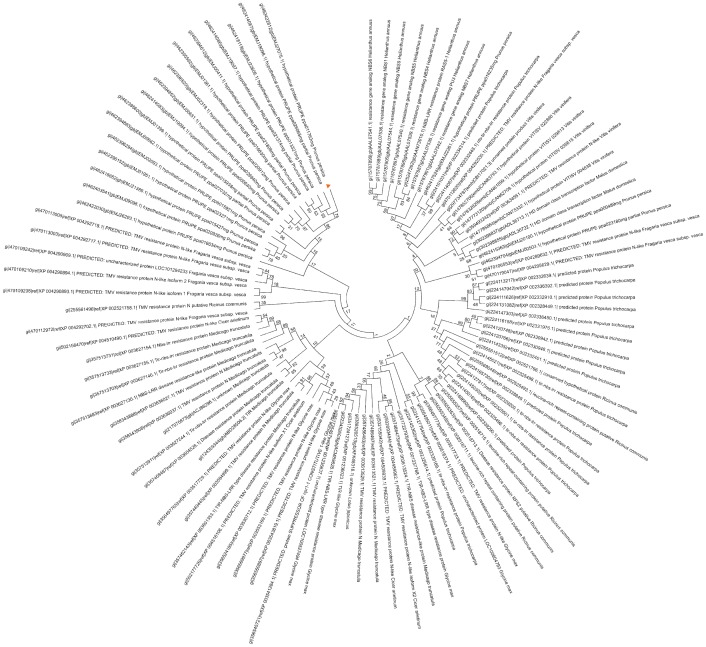
Evolutionary relationships of taxa. The bootstrap consensus tree inferred from 1000 replicates is taken to represent the evolutionary history of the taxa analyzed. Branches corresponding to partitions reproduced in less than 50% bootstrap replicates are collapsed. The evolutionary distances were computed using the Poisson correction method and are in the units of the number of amino acid substitutions per site. The analysis involved 100 amino acid sequences. All positions containing gaps and missing data were eliminated. There were a total of 85 positions in the final dataset.

SNP marker UCD_SNP_641 (scaffold 1:9010281) is located in the haplotype for QTL1.1 and explains 26.6% of the phenotypic variance. It is associated with a 1293 nucleotides gene with high similarity to the N-gene (ppa011763 m.g) for Tobacco mosaic virus (TMV) resistance in tobacco. The N gene was cloned by Whitham et al. [29], and it encodes a TIR-NB-LRR R protein [29]. The plant immune systems model proposed that the recognition to the pathogen molecules in plants is mediated by NB-LRR domains [30]. However, the specific nature of the recognition signal remain unresolved [31]. More recently, biochemical cell fraction and immnunoprecipitation combined with confocal microscopy was used to study the relation between the N domain and p50 (50-kDa helicase domain of the Tobacco mosaic virus). A new complex model for p50 recognition was proposed where the TIR domain of the N gene is necessary and sufficient for association with the p50 Avr elicitor [31], [32]. Given the results of this new research, and the absence of NBS and LRR domains in our transcript sequence of ppa011763m, we think that host recognition of *M. fructicola* in peach can be mediated by this candidate gene, activating the effector-triggered immunity (ETI) response [30]. A phylogenetic analysis was conducted using a data set from the alignment (100 sequences) of the amino acid sequences of our ppa011763m gene together with several paralogous proteins and other protein sequences, related to our gene but from other species. This analysis showed that out candidate forms a different clade from all other paralogous genes, and is supported by a strong (74%) bootstrap support value (Figure 5). The topology observed implies that this gene family has diversified recently (multiple paralogous duplications), perhaps only within the genus. Absence of more similar sequences in *Malus domestica* or *Fragaria vesca* implies that gene duplication occurred after divergence between *Prunus* and *Malus* lineages. Other *Prunus* transcripts similar to this sequence bear the NBS-LRR domains, therefore domain deletion occurred recently.

The SNP marker UCD_SNP_1472 is associated with the receptor-like protein kinase ANXUR2 (ppa026453m), a protein that controls pollen tube behavior by directing rupture at proper timing to release the sperm cell [33]. Many plant receptor-like kinases (RLKs) have been functionally characterized and shown to be involved in a range of environmental and developmental responses. About a dozen of these, the non-RD kinases (e.g., Xa21, FLS2, EFR), have been shown to be important as pattern recognition receptors in PAMP-triggered immune responses to pathogens [34], [35]. This gene (scaffold 1: 8,812,690bp) is physically close to ppa011763m (scaffold 1: 9006452) in the peach genome. According to the zigzag model proposed [30], our ppa026453m could be a candidate gene for peach to trigger PAMP-triggered immunity (PTI) response for *M. fructicola* in peach. PTI have been observed to be involved in signaling through Ca2+ and H+ influx, early accumulation of reactive oxygen species (ROS), the mitogen-activated protein kinases (MAPKs), etc. [36]. The observed SNP marker UCD_SNP_1027 located in the QTL1.2 (59cM), which explained 14.20% of the observed phenotypic variance with the three-years data set and 25.30% with two-years data set, was associated with MPK12_ORYSJ (ppa003297m), a gene with similarity to a rice mitogen-activated protein kinase (MAPK kinase 1) ([37]; Table 3). In rice, MAP kinase 1 has been induced by hydrogen peroxide, salicylic acid, jasmonic acid, ethylene, abscisic acid, infection with rice blast fungus (*Magnaporthe oryzae* (T.T. Hebert) M.E. Barr), an elicitor from *M. oryzae*, and wounding [37].

From the other candidate genes associated with brown rot resistance (Table 3), the SNP marker UCD_SNP_1010 (scaffold 1:10096628) is associated with the bidirectional sugar transporter SWEET8 (or ruptured pollen grain1) (ppa022820m). This protein mediates both low-affinity uptake and efflux of sugar across the plasma membrane and is required for microspore cell integrity and primexine pattern formation. Interestingly, this transporter is induced in plants by the pathogenic bacterium *Pseudomonas syringae* Van Hall pv. *tomato* [38]. The UCD_SNP_800 (scaffold 1∶30152386) explained 15% of the phenotypic variance and was associated with ppa012705m, a major latex protein (MLP). The MLP-like protein 329 (ppa012705m) constitutes a protein family that was identified in the latex of opium poppy (*Papaver somniferum* L.) [39]. MLP's are found only in plants and have 24 identified members in *Arabidopsis thaliana* L. alone with representatives in other plants such as peach, strawberry, melon, cucumber, and soybean. While the function of the MLPs is unknown, they have been associated with fruit and flower development and in pathogen defense responses [40].

The SNP marker UCD_SNP_366 is associated with putative pectinesterase/pectinesterase inhibitor 28 (ppa003441m). The pectinesterase (PME) is located in the cellular membrane, catalyzing the de-esterification of pectin, one of the main components of the plant cell wall, to pectate and methanol. Plant PMEs have been involved with response to fungus pathogens [41], being regulated by pectinesterase inhibitors (PMEIs), which are ineffective against microbial enzymes [42]. A model to describe the regulation mechanism of PME and the interaction between PME and PMEIs has been recently published [43]. In tobacco, fungal PME from *Aspergillus niger* van Tieghem promotes dwarfism in plants [44]. The secretion of a set of pectin-degrading enzymes including PME, polygalacturonase and pectin lyase by this fungus, promotes the decomposition of plant cell during infection and may promote nutrients acquisition from the host [45]. A similar infection pattern could be expected for *M. fructicola* colonization of peach fruits, as similar treatments are used to eliminate both fungi [46]. This candidate gene (ppa003441m) could be the gene responsible for peach fruit infection during the pathogen attack.

SNP effects associated with candidate genes have been linked to disease response QTLs and SNP markers for those QTLs. Although the functional significance of these candidate genes in the *M. fructicola*-peach interaction remains to be established, the discovery of their association with the variation in fruit disease resistance by the analysis used here is intriguing.

## Conclusion

The interaction and expression of SNP-effected proteins could explain the variation observed in each individual and facilitate understanding of gene regulatory networks for brown rot resistance. The combination of classical QTL analysis, genomic annotation in peach, and bioinformatics allowed us to obtain a list of new candidate genes that may lead to a mechanistic understanding of brown rot resistance in peach. The location of the candidate genes, mapped from QTLs, compared to homeologous, and located on the peach 1.0 draft will permit the use of specific gene modification technologies, such as TALENs [47], to alter gene action at specific loci. We have identified at least two potential candidate genes, ppa011763m (TIR domain) and ppa026453m, that may be the genes primarily responsible for *M. fructicola* recognition in peach, activating both PTI and ETI responses. Manipulation of both genes should help geneticists to develop new peach cultivars with brown rot resistance.
